# Associations of maternal 25-hydroxyvitamin D in pregnancy with offspring cardiovascular risk factors in childhood and adolescence: findings from the Avon Longitudinal Study of Parents and Children

**DOI:** 10.1136/heartjnl-2013-303678

**Published:** 2013-10-14

**Authors:** Dylan M Williams, Abigail Fraser, William D Fraser, Elina Hyppönen, George Davey Smith, John Deanfield, Aroon Hingorani, Naveed Sattar, Debbie A Lawlor

**Affiliations:** 1 MRC Centre for Causal Analyses in Translational Epidemiology, School of Social and Community Medicine, University of Bristol, Bristol, UK; 2 Norwich Medical School, University of East Anglia, Norwich, UK; 3 Centre for Paediatric Epidemiology and Biostatistics, and Medical Research Council Centre of Epidemiology for Child Health, University College London Institute of Child Health, London, UK; 4 Institute of Child Health, University College London, London, UK; 5 Genetic Epidemiology Group, Department of Epidemiology and Public Health, University College London, London, UK; 6 BHF Glasgow Cardiovascular Research Centre, University of Glasgow, Glasgow, UK

**Keywords:** LIPIDS

## Abstract

**Objective:**

Lower maternal vitamin D status in pregnancy may be associated with increased offspring cardiovascular risk in later life, but evidence for this is scant. We examined associations of maternal total 25-hydroxyvitamin D (25(OH)D) in pregnancy with offspring cardiovascular risk factors assessed in childhood and adolescence.

**Design:**

A longitudinal, prospective study.

**Setting:**

The study was based on data from mother–offspring pairs in the Avon Longitudinal Study of Parents and Children (ALSPAC), a UK prospective population-based birth cohort (N=4109).

**Outcome measures:**

Offspring cardiovascular risk factors were measured in childhood (mean age 9.9 years) and in adolescence (mean age 15.4 years): blood pressure, lipids, apolipoproteins (at 9.9 years only), glucose and insulin (at 15.4 years only), C reactive protein (CRP), and interleukin 6 (at 9.9 years only) were measured.

**Results:**

After adjustments for potential confounders (maternal age, education, body mass index (BMI), smoking, physical activity, parity, socioeconomic position, ethnicity, and offspring gestational age at 25(OH)D sampling; gender, age, and BMI at outcome assessment), maternal 25(OH)D was inversely associated with systolic blood pressure (−0.48 mm Hg difference per 50 nmol/L increase in 25(OH)D; 95% CI −0.95 to −0.01), Apo-B (−0.01 mg/dL difference; 95% CI −0.02 to −0.001), and CRP (−6.1% difference; 95% CI −11.5% to −0.3%) at age 9.9 years. These associations were not present for risk factors measured at 15.4 years, with the exception of a weak inverse association with CRP (−5.5% difference; 95% CI −11.4% to 0.8%). There was no strong evidence of associations with offspring triglycerides, glucose or insulin.

**Conclusions:**

Our findings suggest that fetal exposure to 25(OH)D is unlikely to influence cardiovascular risk factors of individuals later in life.

## Introduction

Low vitamin D status, assessed by circulating total 25-hydroxyvitamin D (25(OH)D), is common in pregnancy.^1^ ^2^ Maternal 25(OH)D diffuses freely across the placenta, and fetal exposure to vitamin D depends solely on concentrations in the mother.^3^ There is increasing evidence that vitamin D status in pregnancy may influence normal fetal growth and development, and influence offspring health outcomes in later life. Recent observational studies have reported associations of low maternal 25(OH)D concentrations or dietary vitamin D intake with lower bone mineral accrual,^4^ ^5^ and increased risk of type 1 diabetes^6^ and wheezing^7^ in offspring. It has also been suggested that lower concentrations of maternal 25(OH)D in pregnancy might be related to increased risk of insulin resistance (measured by the homeostasis model of assessment-insulin resistance; HOMA-IR), and hence cardiovascular disease, in offspring in later life.^8^

There are several plausible pathways by which maternal 25(OH)D in pregnancy may relate to future cardiovascular health of offspring. First, some,^9–11^ though not all,^12^ ^13^ studies have shown an association of lower 25(OH)D concentration in pregnancy with maternal risk of pre-eclampsia and with low birth weight/risk of a small for gestational age birth in their infants, and these have been associated with future cardiovascular risk in offspring.^14–16^ Secondly, a number of studies have reported associations of lower circulating 25(OH)D with adverse cardiovascular risk factors in children, adolescents, and adults.^17^ ^18^ It is therefore possible that maternal 25(OH)D in pregnancy will be associated with offspring cardiovascular risk factors because maternal and offspring 25(OH)D are correlated due to shared environmental and genetic determinants of 25(OH)D (ie, maternal 25(OH)D in pregnancy will relate to offspring outcomes at least in part because it reflects the child's own concentrations). Thirdly, it is possible that variation in exposure to intrauterine concentrations of 25(OH)D programmes fetal development and influences arterial structure and metabolic processes that affect future cardiovascular health. However, only two small studies have examined associations of maternal 25(OH)D concentrations during pregnancy and cardiovascular disease risk factors of offspring to date.^8^ ^19^ The small sample sizes of both of these studies (with 178 and 539 maternal–offspring pairs) could have limited their ability to detect associations.

Our aim was to examine associations of maternal 25(OH)D concentrations measured in pregnancy with a range of offspring cardiovascular risk factors (blood pressure, lipids, apolipoproteins (Apo-A1, Apo-B), fasting glucose and insulin, C reactive protein (CRP), and interleukin 6 (IL6)) measured during childhood (mean age 9.9 years) and again in adolescence (mean age 15.4 years).

## Methods

### Participants

**Figure 1:**
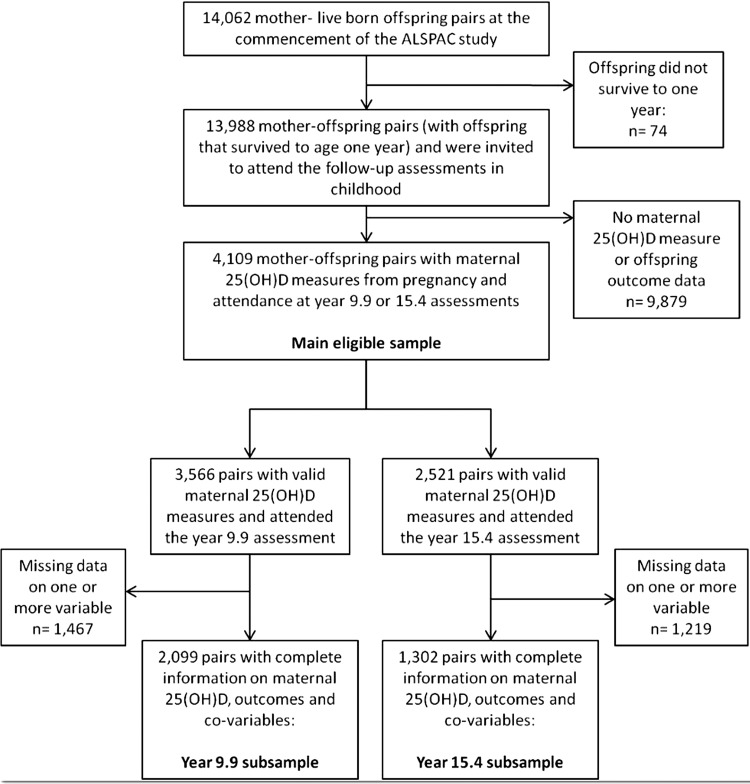
Flow chart of study participants. 25(OH)D, 25-hydroxyvitamin D; ALSPAC, Avon Longitudinal Study of Parents and Children.

The Avon Longitudinal Study of Parents and Children (ALSPAC) is a prospective birth cohort that recruited pregnant women (N=14 541) living within the former county of Avon, South West England. Women with an expected delivery date between 1 April 1991 and 31 December 1992 were eligible to be included. Study details have been published,^20^ ^21^ and are found online at http://www.bristol.ac.uk/alspac/. A total of 13 988 live born children who survived past age 1 year have been followed up alongside their mothers with questionnaires during early childhood, and at regular assessments from age 7. Ethical approval was granted by the ALSPAC Law and Ethics Committee and the local research ethics committee. Written informed consent/assent was obtained from both parents/guardians and the children. For this study, we used measures of 25(OH)D concentrations from blood samples collected from mothers during pregnancy as part of their routine follow-up, and offspring cardiovascular risk factors measured when the offspring attended the year 9.9 and 15.4 year follow-up assessments. Our eligible sample consists of 4109 maternal–offspring pairs with a maternal 25(OH)D measure from pregnancy and offspring cardiovascular risk factors measured at mean age 9.9 or 15.4 years (see figure 1).

### Measures

Details of 25(OH)D assaying, and measurements of outcomes and co-variables, are included in the online supplementary material.

### Statistical analysis

A large proportion of our sample (N=3169; 77.1%) had maternal 25(OH)D_2_ concentrations at or below the assay detection limit (1.25 nmol/L). These were assigned a value of 0 nmol/L. A measure of total 25(OH)D was then calculated from the sum of 25(OH)D_2_ and 25(OH)D_3_ and all associations are of maternal total 25(OH)D with offspring outcomes. 25(OH)D was adjusted for season of sampling, as previously described.^18^ Briefly, 25(OH)D was modelled against the date of blood sampling using linear regression with trigonometric sine and cosine functions, and residuals of regression models were used as season-adjusted 25(OH)D in main analyses.

To test the strength of linear associations between the 25(OH)D measures (unadjusted and season-adjusted maternal 25(OH)D, and unadjusted and season-adjusted offspring 25(OH)D), we calculated Pearson correlation coefficients for pairs of measures.

Multivariable linear regression models were used to examine associations of maternal 25(OH)D with cardiovascular risk factors, and to adjust for potential confounding and mediating factors. Regression coefficients and 95% CI were formatted to show mean differences in outcomes per 50 nmol/L increase in 25(OH)D. Coefficients for log-transformed outcomes (triglycerides, insulin, CRP, and IL6) were expressed in terms of relative percent change per 50 nmol/L increase in 25(OH)D, by reformatting ratios of geometric means and 95% CIs.

We conducted several multivariable linear regression models for each exposure-outcome association. In model 1, associations were adjusted for maternal age at delivery, offspring gender, gestational age at 25(OH)D sampling, age at the year 9.9 or 15.4 assessments, parity, maternal education, household socioeconomic position, ethnicity, maternal pre-pregnancy body mass index (BMI), smoking and physical activity in pregnancy, and offspring BMI at the year 9.9 or 15.4 assessment. We included adjustment for offspring BMI because maternal 25(OH)D is inversely associated with maternal BMI, a mother's BMI may relate to her child's BMI, and offspring BMI is associated with their cardiovascular risk factors. As such, offspring BMI could lie on the confounding pathway. In model 2, we additionally adjusted for potential mediation of associations by offspring 25(OH)D measured in childhood. Model 3 included adjustments for confounders as in model 2, and additional adjustments for potential mediation by gestational hypertension, pre-eclampsia, gestational diabetes mellitus or glycosuria during pregnancy, and birth weight. Possible non-linearity of associations between exposures and outcomes was tested by examining fractional polynomial statistics and interpreting graphical plots.^22^

In addition to examining linear associations, we also conducted multivariable regression analyses examining mean differences of cardiovascular risk factors in offspring with maternal 25(OH)D <50 nmol/L and in offspring with maternal 25(OH)D between 50–75 nmol/L, compared to risk factors in offspring with maternal 25(OH)D > 75 nmol/L.^2^

### Missing data

There were proportions of our eligible sample who had missing data on one or more variable used to examine associations with cardiovascular risk factors at 9.9 years (N=2010; 48.9%) and 15.4 years (N=2807; 68.3%). To address this, we used multivariate multiple imputation to impute missing information on outcomes and covariables for otherwise eligible maternal–offspring pairs with valid maternal 25(OH)D measures from pregnancy and who had attended the year 9.9 or 15.4 assessments. This approach involves switching regression, using the multivariate imputation by chained equations function in Stata.^23^ Twenty cycles of regression switching were used, and estimates of results were averaged across the 20 imputed datasets according to Rubin's rules.^23^ Main analyses were conducted using these datasets.

### Additional analyses

We repeated main analyses using maternal 25(OH)D unadjusted for season of sampling as an exposure. We also repeated main analyses excluding participants whose CRP values suggested acute inflammation (CRP > 6 mg/L).

We tested associations for interactions between maternal 25(OH)D concentrations and trimester of sampling, and examined results after stratifying by trimester.

We also conducted analyses in the subsamples of participants with complete information on maternal 25(OH)D, co-variables and offspring cardiovascular risk factors at mean age 9.9 years (N=2099) and 15.4 years (N=1302).

Given that non-high density lipoprotein cholesterol (non-HDL-C: total cholesterol minus HDL-C) has been implicated as being more strongly associated with cardiovascular events than separate lipid components alone,^24^ we also repeated analyses including non-HDL-C as an outcome. Since a previous study has reported an inverse association of maternal 25(OH)D in pregnancy with offspring insulin resistance as measured by HOMA-IR,^8^ we also repeated analyses with this measure as an outcome, calculated using the standard formula.^25^

## Results

Characteristics of ALSPAC mothers and offspring in the eligible sample, along with characteristics of those who were excluded because of missing data, are shown in online supplemental tables S1 and S2.

Table 1 shows characteristics of ALSPAC mothers and offspring according to categories of maternal 25(OH)D in pregnancy. Maternal age at 25(OH)D sampling in pregnancy, socioeconomic position, maternal education, physical activity, percentage who had never smoked during pregnancy, gestational age at 25(OH)D sampling, birth weight, and offspring 25(OH)D all increased linearly from lower to higher maternal 25(OH)D categories. Parity, percentage of non-white European ethnicity, and percentage who smoked throughout pregnancy all decreased across categories of maternal 25(OH)D. Of risk factors measured at mean age 9.9 years, HDL-C and Apo-A1 increased across low to high categories of maternal 25(OH)D, while diastolic blood pressure (DBP), low density lipoprotein cholesterol (LDL-C) and Apo-B decreased across the categories. Similar trends were observed for LDL-C, HDL-C, and CRP measured at mean age 15.4 years. No trends with other risk factors were observed.

**Table 1:** Characteristics of ALSPAC mothers and offspring by categories of maternal 25(OH)D concentration pregnancy (% or mean and 95% CI; the column marked ‘N’ denotes the number of participants in the analysis sample with available data for each variable)

	N	25(OH)D<25 nmol/L		25(OH) D=25–49.9 nmol/L		25(OH)D=50–75 nmol/L		25(OH)D>75 nmol/L		p Value
*Maternal characteristics*										
Maternal age at delivery	4031	27.5	(26.7 to 28.2)	28.6	(28.3 to 28.8)	28.8	(28.6 to 29.1)	29.4	(29.2 to 29.6)	<0.001
% Parity	4109									
0		55.8		47.0		45.9		42.1		0.001
1		32.7		34.4		34.2		38.4		0.02
2		9.5		13.3		15.0		14.4		0.15
3		2.0		3.2		4.0		4.5		0.05
4 or 5		0.0		2.0		0.9		0.6		0.02
% Non-white European ethnicity	4109	8.5		2.5		1.2		0.9		<0.001
% Socioeconomic position	4109									
I/II		51.9		56.3		59.6		61.7		<0.001
III (non-manual)		28.2		26.2		25.7		24.0		0.35
III (manual)		14.8		12.8		10.6		10.7		0.10
IV/V		5.2		4.8		4.2		3.7		0.22
Maternal education (% attended university)	3868	12.1		14.9		15.1		16.7		0.09
Pre-pregnancy BMI	3620	22.7	(22.1 to 23.3)	22.9	(22.7 to 23.1)	22.9	(22.7 to 23.1)	22.7	(22.5 to 22.9)	0.20
Maternal smoking (%)	4109									
Never		72.5		75.0		80.7		83.4		<0.001
Before or during first trimester		4.0		6.7		5.7		5.4		0.44
Throughout pregnancy		23.5		18.3		13.6		11.3		<0.001
Maternal physical activity in pregnancy (MET)*	3268	9.9	(8.2 to 12.0)	11.3	(10.5 to 12.1)	12.0	(11.2 to 12.8)	12.6	(11.9 to 13.4)	0.003
% Gestational hypertension	3947	15.5		14.4		15.3		13.0		0.23
% Pre-eclampsia	4013	2.6		1.8		1.4		1.6		0.42
% Gestational diabetes	3951	0.0		0.7		0.8		0.5		0.98
% Glycosuria in pregnancy	4109	2.5		3.2		2.8		2.4		0.31
*Offspring characteristics*										
% male	4109	54.4		49.5		51.9		52.2		0.39
Gestational age at 25(OH)D sampling (weeks)	4109	24.0	(22.3 to 25.7)	23.4	(22.8 to 24.0)	23.7	(23.1 to 24.3)	25.7	(25.1 to 26.2)	<0.001
Birth weight (kg)	3982	3.3	(3.2 to 3.4)	3.4	(3.4 to 3.5)	3.4	(3.4 to 3.5)	3.5	(3.5 to 3.5)	<0.001
Age at year 9.9 assessment (years)	3566	9.94	(9.89 to 10.00)	9.87	(9.85 to 9.89)	9.84	(9.82 to 9.86)	9.86	(9.85 to 9.88)	0.16
Age at year 15.4 assessment (years)	2521	15.51	(15.44 to 15.57)	15.45	(15.42 to 15.47)	15.44	(15.42 to 15.46)	15.44	(15.42 to 15.46)	0.21
BMI at year 9.9 assessment (kg/m2)	3525	17.6	(17.1 to 18.0)	17.7	(17.5 to 17.8)	17.6	(17.4 to 17.8)	17.7	(17.5 to 17.8)	0.87
BMI at year 15.4 assessment (kg/m2)	2497	21.4	(20.7 to 22.1)	21.3	(21.1 to 21.6)	21.3	(21.1 to 21.6)	21.2	(20.9 to 21.4)	0.27
Childhood 25(OH)D (nmol/L)	4099	22.9	(21.4 to 24.3)	24.4	(23.8 to 24.9)	25.3	(24.8 to 25.9)	26.6	(26.1 to 27.0)	<0.001
*Year 9.9 risk factors*										
SBP (mm Hg)	3525	102.9	(101.4 to 104.5)	102.9	(102.3 to 103.5)	102.5	(101.9 to 103.0)	102.3	(101.8 to 102.8)	0.10
DBP (mm Hg)	3527	57.6	(56.5 to 58.7)	57.7	(57.3 to 58.1)	57.3	(56.9 to 57.6)	57.2	(56.8 to 57.5)	0.05
Triglycerides (mmol/L)†	2770	0.99	(0.91 to 1.08)	1.05	(1.02 to 1.08)	1.03	(1.00 to 1.06)	1.03	(1.00 to 1.06)	0.80
LDL-C (mmol/L)	2770	2.39	(2.28 to 2.51)	2.37	(2.32 to 2.41)	2.32	(2.28 to 2.36)	2.31	(2.28 to 2.35)	0.05
HDL-C (mmol/L)	2770	1.38	(1.32 to 1.44)	1.38	(1.36 to 1.40)	1.41	(1.39 to 1.43)	1.41	(1.39 to 1.43)	0.02
Apo-A1 (mg/dL)	2770	1.33	(1.29 to 1.37)	1.35	(1.34 to 1.37)	1.37	(1.35 to 1.38)	1.37	(1.35 to 1.38)	0.04
Apo-B (mg/dL)	2770	0.61	(0.58 to 0.63)	0.60	(0.59 to 0.61)	0.59	(0.58 to 0.59)	0.58	(0.57 to 0.59)	0.002
CRP (mg/L)†	2388	0.26	(0.21 to 0.33)	0.29	(0.27 to 0.31)	0.26	(0.24 to 0.28)	0.26	(0.24 to 0.28)	0.10
IL6 (pg/mL)†	2760	0.86	(0.73 to 1.01)	0.88	(0.83 to 0.94)	0.83	(0.78 to 0.88)	0.82	(0.78 to 0.87)	0.12
*Year 15.4 risk factors*										
SBP (mm Hg)	2388	122.6	(120.3 to 125.0)	122.7	(121.8 to 123.5)	122.7	(122.0 to 123.5)	123.5	(122.7 to 124.2)	0.15
DBP (mm Hg)	2388	67.5	(65.7 to 69.4)	67.5	(66.8 to 68.1)	67.2	(66.6 to 67.8)	67.7	(67.2 to 68.3)	0.55
Triglycerides (mmol/L)†	1760	0.77	(0.71 to 0.85)	0.77	(0.75 to 0.80)	0.77	(0.75 to 0.79)	0.76	(0.74 to 0.78)	0.33
LDL-C (mmol/L)	1760	2.16	(2.02 to 2.29)	2.11	(2.06 to 2.16)	2.08	(2.03 to 2.12)	2.07	(2.02 to 2.11)	0.12
HDL-C (mmol/L)	1760	1.25	(1.17 to 1.32)	1.27	(1.24 to 1.30)	1.27	(1.25 to 1.30)	1.30	(1.27 to 1.32)	0.07
Glucose (mmol/L)	1760	5.22	(5.12 to 5.32)	5.17	(5.13 to 5.20)	5.21	(5.17 to 5.24)	5.21	(5.18 to 5.24)	0.15
Insulin (IU/L)†	1757	9.18	(8.13 to 10.35)	8.77	(8.40 to 9.17)	8.94	(8.58 to 9.31)	8.81	(8.49 to 9.14)	0.83
CRP (mg/L)†	1760	0.64	(0.49 to 0.84)	0.52	(0.47 to 0.57)	0.52	(0.48 to 0.57)	0.44	(0.41 to 0.48)	0.001

*MET, metabolic equivalent.

†Geometric means.

25(OH)D, Total 25-hydroxyvitamin D; ALSPAC, Avon Longitudinal Study of Parents and Children; Apo-A1, apolipoprotein-A1; Apo-B, apolipoprotein-B; BMI, body mass index; CRP, C reactive protein; DBP, diastolic blood pressure; HDL-C, high density lipoprotein cholesterol; IL6, interleukin 6; LDL-C, low density lipoprotein cholesterol; SBP, systolic blood pressure.

Online supplemental table S3 shows correlations of unadjusted and season-adjusted maternal 25(OH)D, and also offspring unadjusted and season-adjusted 25(OH)D sampled at mean age 9.8 years. There were weak positive correlations of unadjusted and season-adjusted maternal 25(OH)D with unadjusted and season-adjusted offspring 25(OH)D (all Pearson's r=0.11 to 0.15; all p<0.001).

**Figure 2:**
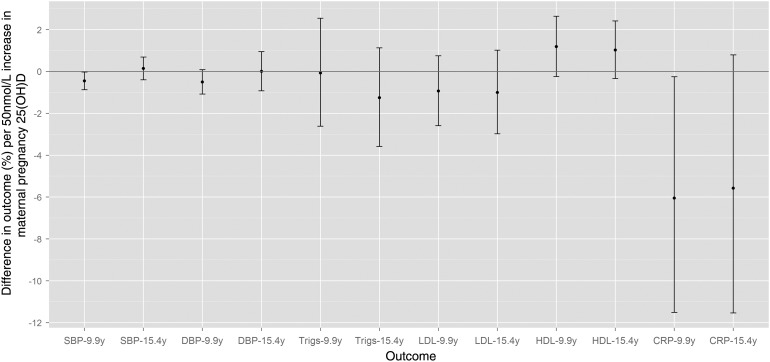
Relative percentage differences (and 95% CI) in offspring outcomes at mean age 9.9 and 15.4 years, per 50 nmol/L of maternal 25-hydroxyvitamin D (25(OH)D) in pregnancy (N=4109). Associations are adjusted for maternal age at delivery, education level, pre-pregnancy body mass index (BMI), smoking and physical activity during pregnancy, parity, socioeconomic position, ethnicity, and offspring gestational age at maternal 25(OH)D sampling, gender, age and BMI at year 9.9 or 15.4 assessment. CRP, C reactive protein; DBP, diastolic blood pressure; HDL, high density lipoprotein; LDL, low density lipoprotein; SBP, systolic blood pressure; Trigs, triglycerides.

Table 2 shows multivariable associations of maternal 25(OH)D with offspring cardiovascular risk factors. In model 1, there were inverse associations of maternal 25(OH)D with systolic blood pressure (SBP), Apo-B and CRP, and weak inverse associations with DBP and IL6, at mean age 9.9 years. At mean age 15.4 years there were no associations with SBP or DBP, but a weak inverse association with CRP was present. Further adjustments for offspring 25(OH)D (model 2) and other potential mediators (model 3) did not substantially change results observed in model 1, although the association of 25(OH)D with CRP at 9.9 years attenuated slightly in model 2. Figure 2 shows the confounder-adjusted associations with risk factors that were measured at both ages, with all results on a scale of percentage difference per 50 nmol/L increase in 25(OH)D. It can be seen that the directions and magnitudes of associations with CRP are similar at both age points.

**Table 2:** Associations of maternal 25(OH)D in pregnancy with offspring cardiovascular risk factors measured at mean age 9.9 and 15.4 years (N=4109; data for individuals with missing values for co-variables were imputed)

	Model 1			Model 2			Model 3		
	Mean difference per 50 nmol/L increase of 25(OH)D	95% CI	p Value	Mean difference per 50 nmol/L increase of 25(OH)D	95% CI	p Value	Mean difference per 50 nmol/L increase of 25(OH)D	95% CI	p Value
9.9 year risk factors									
SBP (mm Hg)	−0.48	(−0.92 to −0.05)	0.03	−0.47	(−0.90 to −0.03)	0.04	−0.44	(−0.87 to −0.01)	0.05
DBP (mm Hg)	−0.29	(−0.63 to 0.04)	0.08	−0.27	(−0.61 to 0.06)	0.11	−0.27	(−0.60 to 0.06)	0.11
Triglycerides (% difference*)	−0.1	(−2.6 to 2.5)	0.96	−0.3	(−2.9 to 2.3)	0.82	−0.1	(−2.6 to 2.5)	0.95
LDL-C (mmol/L)	−0.02	(−0.06 to 0.02)	0.29	−0.02	(−0.06 to 0.01)	0.24	−0.02	(−0.06 to 0.02)	0.29
HDL-C (mmol/L)	0.02	(−0.003 to 0.03)	0.10	0.01	(−0.005 to 0.03)	0.14	0.02	(−0.003 to 0.03)	0.10
Apo-A1 (mg/dL)	0.00	(−0.01 to 0.02)	0.58	0.00	(−0.01 to 0.01)	0.88	0.00	(−0.01 to 0.02)	0.56
Apo-B (mg/dL)	−0.01	(−0.02 to −0.001)	0.03	−0.01	(−0.02 to −0.001)	0.03	−0.01	(−0.02 to −0.001)	0.03
CRP (% difference*)	−6.1	(−11.5 to −0.3)	0.04	−5.5	(−11.1 to 0.5)	0.07	−6.0	(−11.4 to −0.2)	0.04
IL6 (% difference*)	−4.6	(−9.3 to 0.3)	0.07	−3.9	(−8.7 to 1.2)	0.13	−4.6	(−9.4 to 0.3)	0.07
15.4 year risk factors									
SBP (mm Hg)	0.15	(−0.51 to 0.82)	0.65	0.13	(−0.53 to 0.80)	0.69	0.22	(−0.44 to 0.87)	0.52
DBP (mm Hg)	0.01	(−0.60 to 0.63)	0.96	0.00	(−0.61 to 0.61)	1.00	0.02	(−0.59 to 0.63)	0.96
Triglycerides (% difference*)	−1.3	(−3.6 to 1.1)	0.30	−1.1	(−3.3 to 1.3)	0.38	−1.2	(−3.5 to 1.2)	0.31
LDL-C (mmol/L)	−0.02	(−0.06 to 0.02)	0.34	−0.02	(−0.06 to 0.02)	0.29	−0.02	(−0.06 to 0.02)	0.35
HDL-C (mmol/L)	0.01	(−0.004 to 0.03)	0.15	0.01	(−0.01 to 0.03)	0.20	0.01	(−0.005 to 0.03)	0.16
Glucose (mmol/L)	−0.01	(−0.03 to 0.02)	0.72	0.00	(−0.03 to 0.03)	0.90	0.00	(−0.03 to 0.02)	0.74
Insulin (% difference*)	−0.1	(−3.6 to 3.6)	0.96	0.5	(−3.0 to 4.1)	0.78	−0.1	(−3.6 to 3.5)	0.96
CRP (% difference*)	−5.6	(−11.5 to 0.7)	0.08	−5.6	(−11.5 to 0.7)	0.08	−5.3	(−11.2 to 0.9)	0.09

Model 1: adjusted for maternal age at delivery, education level, pre-pregnancy BMI, smoking and physical activity during pregnancy, parity, socioeconomic position, ethnicity, and offspring gestational age at maternal 25(OH)D sampling, gender, age and BMI at year 9.9 or 15.4 assessment.

Model 2: as model 1 plus offspring 25(OH)D in childhood.

Model 3: as model 1 plus gestational hypertension, pre-eclampsia, gestational diabetes or glycosuria in pregnancy, and birth weight.

*Outcomes were log transformed: results represent relative percent differences in outcomes per 50 nmol/L increase of 25(OH)D.

25(OH)D, Total 25-hydroxyvitamin D; Apo-A1, Apolipoprotein-A1; Apo-B, Apolipoprotein-B; BMI, body mass index; CRP, C reactive protein; DBP, diastolic blood pressure; HDL-C, high-density lipoprotein cholesterol; IL6, interleukin 6; LDL-C, low density lipoprotein cholesterol; SBP, systolic blood pressure.

Online supplemental table S4 shows mean differences in cardiovascular risk factors in offspring whose mothers had 25(OH)D from 50–75 nmol/L or 25(OH)D<50 nmol/L in pregnancy, compared to those whose mothers had 25(OH)D>75 nmol/L. On average, Apo-B at 9.9 years was higher in offspring with maternal 25(OH)D concentrations <50 nmol/L or 50–75 nmol/L compared to those with maternal 25(OH)D>75 nmol/L. This difference was small but followed a dose-response pattern. HDL-C was also lower, and CRP higher, at 15.4 years in those in lower maternal 25(OH)D categories compared to those with maternal 25(OH)D>75 nmol/L. Further adjustments for potential mediators produced similar results to model 1 for all risk factor associations (model 2 data shown in online supplemental table S5; model 3 data available on request).

There was no evidence that any association deviated from linearity (all fractional polynomial p≥0.15). There was also limited evidence of interactions between trimester of pregnancy in which 25(OH)D was sampled and associations of season-adjusted maternal 25(OH)D with offspring cardiovascular risk factors. The exception was the association with LDL-C at age 9.9 years (p for interaction=0.02; all other p≥0.24). There was an inverse association of LDL-C with maternal 25(OH)D sampled in trimester 1, but not with 25(OH)D sampled in trimesters 2 or 3.

In general, the directions and magnitudes of associations of maternal 25(OH)D unadjusted for season of sampling with cardiovascular risk factors were very similar to results of main analyses (see online supplemental table S6). The results of analyses conducted on complete case subsamples are shown in online supplemental table S7. Results were similar to those of analyses conducted on imputed datasets; the main notable difference was an absence of an inverse association of maternal 25(OH)D with SBP measured at 9.9 years, and the presence of an inverse association with LDL-C at 15.4 years.

Removing participants with high CRP values (>6 mg/L) did not appreciably change associations for inflammatory markers, although the inverse association of maternal 25(OH)D with CRP at 15.4 years strengthened. In the confounder-adjusted model, there was a −6.2% difference in CRP per 50 nmol/L increase in season-adjusted maternal 25(OH)D (95% CI −11.6 to −0.4).

Analyses using non-HDL-C and HOMA-IR as outcomes were consistent with those using LDL-C and fasting insulin, respectively (data available on request).

## Discussion

This study provides limited evidence to support the hypothesis that intrauterine 25(OH)D exposure influences cardiovascular risk factors measured in childhood and adolescence. We found inverse associations between maternal 25(OH)D measured in pregnancy and offspring CRP measured in childhood and adolescence. We also found evidence for inverse associations of maternal 25(OH)D with offspring SBP and Apo-B at 9.9 years, although there was no association with SBP measured at 15.4 years (Apo-B measurements at 15.4 years were not available). There was no consistent evidence for associations with the following cardiovascular risk factors measured at either assessment: DBP, lipids, IL6, and fasting glucose and insulin (the latter two risk factors measured only in adolescence).

Two small existing studies have examined maternal 25(OH)D concentrations in pregnancy in relation to offspring cardiovascular risk factors in childhood.^8^ ^19^ In the current study, there was some evidence for an inverse association of maternal 25(OH)D with offspring SBP in childhood, which contrasts with the two previous studies of this nature. However, our results also suggest that associations with blood pressure are not present after childhood. The lack of consistent associations with offspring blood pressure at both of the age points counters the hypothesis that exposure to maternal 25(OH)D in pregnancy helps to programme lifelong blood pressure in offspring. Fasting insulin at 9.5 years was higher in Indian offspring of mothers with 25(OH)D<50 nmol/L in pregnancy than those whose mothers had 25(OH)D over 50 nmol/L (N=578),^8^ but we found no similar relation of maternal 25(OH)D to fasting insulin of offspring at 15.4 years. Although we cannot rule out potential ethnic differences in associations, the findings of this study suggest that the effects of in utero 25(OH)D on fetal pancreatic development, β-cell function or mechanisms for glucose homeostasis are limited, and maternal 25(OH)D in pregnancy is unlikely to be a key aetiological risk factor for the future development of type-2 diabetes in offspring.

In the Indian Mysore Parthenon cohort, higher maternal 25(OH)D status in pregnancy was associated with lower HDL-C in males at 9.5 years (but not females).^8^ In our study, higher maternal 25(OH)D in pregnancy was only associated with cardioprotective levels of Apo-B, and not Apo-A1 or lipoproteins.

Neither of the previous studies had examined associations of maternal 25(OH)D with offspring inflammatory markers, and to our knowledge this is the first study to report inverse associations of maternal 25(OH)D in pregnancy with CRP values in offspring. Although standard errors for associations were large (and some confidence intervals included the null), the point estimates for these associations were strong, with CRP values in adolescence decreasing by approximately 5.6% per 50 nmol/L of 25(OH)D in pregnancy.

If such associations are causal and not a result of residual confounding, it is unclear how fetal exposure to 25(OH) D may affect chronic inflammation (via supply of the active molecule 1,25(OH)_2_D). The vitamin D system may help to increase the expression of T helper type 2 (Th2) cells and inhibit T helper type 1 (Th1) cell differentiation during pregnancy.^26^ Over-expression of Th1 relative to Th2 (along with changes to circulating cytokines produced by these cells) is thought to increase the risk of conditions associated with adverse immunomodulation, such as pre-eclampsia.^27^ It is possible that the determination of the T cell balance in pregnancy may also programme long term immune responses in offspring. In line with this hypothesis, higher maternal vitamin D intake or neonatal 25(OH)D status has been associated with reduced risk of childhood wheezing (which may depend on improved inflammatory response) in offspring.^7^ ^28^ However, further studies are necessary to increase our understanding of mechanisms linking in utero 25(OH)D exposure to lifelong inflammatory response.

### Strengths and limitations

Our study has important strengths. It is several times larger than the two existing studies of a similar nature, so we have greater power to detect small but real associations. It is the first to have compared associations of maternal 25(OH)D with offspring cardiovascular risk factors measured at time points in both childhood and adolescence. We were also able to examine whether associations were due to shared familial characteristics that may influence 25(OH)D concentrations of both mothers and offspring, rather than being due to intrauterine effects of maternal 25(OH)D alone. Finally, our analyses were conducted on a large, non-select general population.

The main limitation of the study is attrition to participation across the course of the study, which is common in longitudinal cohorts. However, although there was statistical evidence for differences in several characteristics between the eligible sample and those excluded because of missing data on maternal 25(OH)D and/or offspring cardiovascular risk factors, these differences were small in magnitude. Furthermore, attrition would only introduce bias if the relationship between maternal 25(OH)D in pregnancy and offspring cardiovascular risk factors was different in those who originally enrolled but had been subsequently excluded, compared to our included sample, which we do not anticipate. Maternal 25(OH)D was assessed using single measures, so regression dilution may have occurred, and reported results could be weaker than true associations. However, 25(OH)D concentrations have been shown to correlate strongly over time, so a single measure of 25(OH)D may serve as an acceptable proxy for overall vitamin D status during the period of exposure measurement (and similarly for childhood vitamin D status).^29^ ^30^

## Conclusions

The concept of increasing maternal 25(OH)D concentration during pregnancy in order to improve non-skeletal health outcomes in offspring is novel, and calls from health practitioners advocating vitamin D supplementation in pregnancy for this purpose may be premature. Although our results suggest the possibility of associations of higher 25(OH)D with healthier concentrations of CRP (and also Apo-B), further prospective studies are required to confirm the findings and experimental studies are required to increase our understanding of potential mechanisms. If findings are replicated elsewhere, randomised controlled trials aimed at increasing maternal 25(OH)D concentrations in pregnancy would be warranted to see if vitamin D supplementation can improve levels of chronic inflammation in offspring.

## Supplementary Material

Web supplement
