# Supplementary material for: Associations of maternal 25-hydroxyvitamin D in pregnancy with offspring cardiovascular risk factors in childhood and adolescence: findings from the Avon Longitudinal Study of Parents and Children
Source: Heart. 2013 Oct 14;99(24):1849–56. doi: 10.1136/heartjnl-2013-303678 (PMC3841764; doi:10.1136/heartjnl-2013-303678)
Supplement: Web supplement [file heartjnl-2013-303678-s1.pdf]

SUPPLEMENTAL MATERIAL

Maternal and offspring 25(OH)D assessment

Maternal circulating 25(OH)D_2 and 25(OH)D_3 were measured on non-fasting blood samples that were taken for routine pregnancy blood tests (residuals from these samples were used to measure 25(OH)D). Samples were initially stored at -20°C and then at -80°C.

25(OH)D assays were performed in 2010 and 2011 after a maximum of 21 years in storage for pregnancy samples, and 12 years for offspring samples. Measurement of both maternal and offspring 25(OH)D concentration were undertaken in the same laboratory using identical procedures. Concentrations were measured with high performance liquid chromatography tandem mass spectrometry using an internal standard in a laboratory meeting the performance target set by the Vitamin D External Quality Assessment Scheme (DEQAS) Advisory Panel for 25(OH)D assays. 25(OH)D_2, 25(OH)D_3 and the deuterated internal standard were extracted from serum samples, following protein precipitation, using Isolute C18 solid phase extraction cartridges. Potential interfering compounds were removed by initial elution with 50% methanol followed by elution of the vitamins using 10% tetrahydrofuran in acetonitrile. Dried extracts were reconstituted prior to injection into a HPLC tandem mass spectrometer in the multiple reaction mode (MRM). The MRM transitions (m/z) used were 413.2 > 395.3, 401.1 > 383.3 and 407.5 > 107.2 for 25(OH)D_2, 25(OH)D_3, and hexa deuterated (OH)D_3 respectively. Coefficients of variation for the assay were <10% across a working range of 2.5 nmol/L to 624nmol/L for both 25(OH)D_2 and 25(OH)D_3.

Here, priority was given to maternal samples taken in the second or third trimesters of pregnancy, in keeping with previous studies of this nature that also used 25(OH)D measures from late pregnancy [1 2]. Most offspring 25(OH)D measures were sampled at the year 9.9

assessment. For associations with cardiovascular risk factors at mean age 15.4 years, adjustment for offspring 25(OH)D concentrations was conducted using samples from the year 11 (N=657) or year 7 (N=669) assessments when year 9.9 samples were not available.

Offspring outcomes

Systolic and diastolic blood pressure (SBP and DBP) were measured at both the 9.9 and 15.4 year assessments using identical protocols and equipment. These were measured twice using a Dinamap 9301 Vital Signs Monitor (Morton Medical, London, UK) with the participant resting, and their arm supported at chest level; the mean of the two readings were used in analyses.

All blood-based outcomes from the 9.9 year assessment were measured on non-fasting blood samples; these were assayed in 2008 after a median of 7.5 years in storage. Participants fasted overnight before attending the 15.4 year assessment if seen in the morning, or for a minimum of 6 hours if seen in the afternoon. Measurements using these samples were assayed within 3 to 12 months of the samples being taken. Cardiovascular risk factors from both assessments were measured at the same laboratory, using the same methods for identical assays.

Lipids and CRP were measured at both assessments. Lipids (total cholesterol, triglycerides and HDL-C) were assessed by modification of the standard Lipid Research Clinics Protocol using enzymatic reagents for lipid determination. LDL-C levels were derived from measures of total cholesterol, triglycerides and HDL-C using the Friedewald calculation [3]. C-reactive protein (CRP) was measured by automated particle-enhanced immunoturbidimetric assay supplied by Roche (Indiana, USA).

Apolipoproteins and IL-6 were measured using samples from the 9.9 year assessment only. Apolipoprotein A1 (Apo-A1) and Apolipoprotein B (Apo-B) were measured by immunoturbidimetric assays (Roche, Indiana, US). Interleukin-6 (IL-6) was measured by ELISA (R&D systems, Abingdon, UK)

Fasting glucose and insulin were measured using samples from the 15.4 year assessment only. Insulin was measured by an ELISA (Mercodia, Uppsala, Sweden) that does not cross-react with proinsulin, and plasma glucose was measured by automated enzymatic (hexokinase) assay using a Hitachi Modular P analyzer (Roche, Indiana, USA). All assay coefficients of variation were <10% across the working ranges of the assays.

Other variables

Information on maternal age at delivery and gestational age of offspring at maternal 25(OH)D sampling, birth weight, and diagnosis of gestational hypertension, preeclampsia, gestational diabetes and/or glycosuria were extracted from obstetric records.

Household socioeconomic position was obtained from questions completed by the mother at the time of recruitment (in early pregnancy) about the mother's and her partners' longest occupation. Responses were classified according to the registrar generals' classification (from I (professionals and skilled managers) to V (unskilled manual workers)). Maternal education was also recorded, as whether or not mothers attended university. Parity and ethnicity were also obtained by self-report from the mother at recruitment; the vast majority of the cohort is defined as white European, so ethnicity was dichotomized as white European origin or not.

Information on height, pre-pregnancy weight, and maternal smoking and physical activity in pregnancy were obtained from questionnaire responses. Pre-pregnancy body mass index (BMI : weight (kg)/height $(m)^2$) was derived from these responses. Maternal smoking in

pregnancy was categorized as never smoked, smoked before pregnancy or throughout the first trimester before stopping, or smoked throughout the entire pregnancy. Physical activity in pregnancy was assessed at 18 weeks of gestation, expressed in average metabolic equivalent (MET) scores, as previously described [4].

Weight and height of offspring at the 9.9 and 15.4 year assessments were measured in light clothing and without shoes, and used to derive BMI. Weight was measured to the nearest 0.1 kg using Tanita scales (Wardworth Ltd, Bolton, UK). Height was measured to the nearest 0.1 cm using a Harpenden stadiometer (Holtain Ltd, Crymych Pembrokeshire, UK).

Supplemental table 1: Characteristics of ALSPAC mothers in the eligible sample, and those excluded because of missing data (i.e. no data on 25(OH)D in pregnancy or offspring outcomes at mean age 9.9 or 15.4 years)

	Excluded participants		Eligible sample		
	N	Mean (SD) or %	N	Mean (SD) or %	*P*
25(OH)D (nmol/L)	4635	65.4 (32.1)	4109	68.1 (32.3)	<0.001
Trimester of 25(OH)D sampling	4635		4109		<0.001
I		24.1		24.3	
II		27.0		21.3	
III		49.0		54.4	
Age at delivery (years)	9673	27.6 (5.1)	4031	28.9 (4.6)	<0.001
% Parity	8850		3880		<0.001
0		44.8		45.2	
1		34.4		35.7	
2		14.4		14.1	
3		4.3		3.9	
4 or 5		2.1		1.1	
% non-white European ethnicity	2971	2.3	3863	1.7	0.07
% socioeconomic position	7570		3698		<0.001
I/II		12.3		15.5	
III (non-manual)		40.7		43.6	
III (manual)		25.7		25.3	
IV/V		21.3		15.6	
Education level (% attended university)	8292	11.6	3868	15.5	<0.001
Pre-pregnancy BMI (kg/m2)	2624	23.0 (3.8)	3620	22.8 (3.7)	0.13
Smoking (%)	8985		3926		<0.001
never		72.1		79.8	
before or during first trimester		7.4		5.8	
throughout pregnancy		20.5		14.5	
Physical activity in pregnancy (MET) *	2760	15.2 (2.9, 25.9)	3732	15.2 (2.9, 24.5)	0.47
% gest. hypertension	9276	14.4	3947	14.2	0.05
% preeclampsia	9498	2.3	4013	1.6	0.01
% gest. diabetes	8258	0.4	3859	0.7	0.02
% glycosuria in pregnancy	8258	3.5	3859	3.0	0.15

25(OH)D, Total 25-hydroxyvitamin D; BMI, Body mass index

In the analysis sample, for co-variables with missing data, the values presented in this table were based on measured data, rather than imputed datasets

* Median and interquartile range presented because of skewed distribution

Supplemental table 2: Characteristics and cardiovascular risk factors of ALSPAC offspring in the eligible sample, and those excluded because of missing data (i.e. no data on 25(OH)D in pregnancy or offspring outcomes at mean age 9.9 or 15.4 years)

	Excluded participants		Eligible sample		
	N	Mean (SD) or %	N	Mean (SD) or %	*P*
% male	3465	49.1	4109	51.4	0.05
Gestational age at 25(OH)D sampling (weeks)	4699	23.8 (10.6)	4109	24.8 (10.6)	<0.001
Birth weight (kg)	3088	3.38 (0.58)	3982	3.45 (0.51)	<0.001
Age at year 9.9 assessment (years)	2961	9.88 (0.31)	3566	9.86 (0.33)	0.03
Age at year 15.4 assessment (years)	2077	15.4 (0.3)	2521	15.4 (0.3)	0.56
BMI at year 9.9 assessment (kg/m^2)	2931	17.7 (2.9)	3525	17.6 (2.8)	0.72
BMI at year 15.4 assessment (kg/m^2)	2044	21.4 (3.5)	2497	21.3 (3.4)	0.12
Childhood 25(OH)D (nmol/L)	3461	63.5 (23.6)	4099	63.4 (23.4)	0.83
Year 9.9 risk factors					
SBP (mmHg)	2927	102.6 (9.2)	3525	102.5 (9.1)	0.82
DBP (mmHg)	2928	57.2 (6.4)	3527	57.4 (6.4)	0.35
Triglycerides (mmol/L) *	2287	1.00 (0.76, 1.36)	2770	1.01 (0.76, 1.38)	0.53
LDL-C (mmol/L)	2287	2.37 (0.59)	2770	2.33 (0.62)	0.04
HDL-C (mmol/L)	2287	1.40 (0.31)	2770	1.40 (0.30)	0.64
Apo-A1 (mg/dL)	2287	1.36 (0.20)	2770	1.36 (0.20)	0.99
Apo-B (mg/dL)	2287	0.60 (0.13)	2770	0.59 (0.13)	0.01
CRP (mg/L)*	2287	0.21 (0.11, 0.56)	2770	0.22 (0.11, 0.53)	0.24
IL-6 (pg/mL)*	2287	0.81 (0.50, 1.45)	2760	0.80 (0.49, 1.41)	0.11
Year 15.4 risk factors					
SBP (mmHg)	1954	123.5 (10.7)	2388	123.0 (10.9)	0.09
DBP (mmHg)	1954	67.5 (8.8)	2388	67.5 (8.7)	0.94
Triglycerides (mmol/L) *	1414	0.76 (0.6, 0.99)	1760	0.74 (0.58, 0.97)	0.23
LDL-C (mmol/L)	1414	2.10 (0.57)	1760	2.08 (0.55)	0.47
HDL-C (mmol/L)	1414	1.29 (0.28)	1760	1.28 (0.30)	0.33
Glucose (mmol/L)	1414	5.22 (0.36)	1760	5.20 (0.39)	0.08
Insulin (IU/L)*	1414	9.3 (6.8)	1757	8.9 (6.7, 12.0)	0.03
CRP (mg/L)*	1414	0.38 (0.22, 0.88)	1760	0.39 (0.22, 0.88)	0.99

25(OH)D, Total 25-hydroxyvitamin D; Apo-A1, Apolipoprotein-A1; Apo-B, Apolipoprotein-B; CRP, C-reactive protein; DBP, diastolic blood pressure; HDL-C, high density lipoprotein cholesterol; IL-6, interleukin 6; LDL-C, low density lipoprotein cholesterol; SBP, systolic blood pressure

In the analysis sample, for co-variables with missing data, the values presented in this table were based on measured data, rather than imputed datasets

* Median and interquartile range presented because of skewed distribution

Supplemental table 3: Correlation coefficients (*r*) of maternal 25(OH)D concentration in pregnancy and offspring 25(OH)D concentration at mean age 9.9 years

	Maternal 25(OH)D in pregnancy	Maternal season-adjusted 25(OH)D in pregnancy	Childhood 25(OH)D	Season-adjusted childhood 25(OH)D
Maternal 25(OH)D in pregnancy	-			
Maternal season-adjusted 25(OH)D in pregnancy	0.93	-		
Childhood 25(OH)D	0.11	0.11	-	
Season-adjusted childhood 25(OH)D	0.13	0.15	0.80	-

25(OH)D, Total 25-hydroxyvitamin D

All *P* for correlations <0.001

Supplemental table 4: Mean differences in offspring cardiovascular risk factors at mean age 9.9 and 15.4 years in those with maternal 25(OH)D = 50 to 75 nmol/L (N=1,284) and those with maternal 25(OH)D < 50nmol/L (N=1,341), compared to those with maternal 25(OH)D > 75 nmol/L (N= 1,484)

	Mean difference in outcomes in those with 25(OH)D=50 to 75 nmol/L vs. those with 25(OH)D >75 nmol/L	95% CI	Mean difference in outcomes in those with 25(OH)D<50 vs. those with 25(OH)D >75 nmol/L	95% CI	*P*†
Year 9.9 risk factors					
SBP (mmHg)	0.34	(-0.40, 1.08)	0.59	(-0.14, 1.31)	0.11
DBP (mmHg)	0.14	(-0.39, 0.66)	0.44	(-0.05, 0.94)	0.08
Triglycerides (% difference)	-0.4	(-4.3, 3.7)	0.5	(-3.4, 4.5)	0.91
LDL-C (mmol/L)	0.01	(-0.04, 0.06)	0.05	(-0.01, 0.10)	0.09
HDL-C (mmol/L)	-0.004	(-0.03, 0.02)	-0.02	(-0.05, 0.01)	0.14
Apo-A1 (mg/dL)	0.00	(-0.02, 0.02)	-0.01	(-0.03, 0.01)	0.26
Apo-B (mg/dL)	0.01	(-0.01, 0.02)	0.02	(0.00, 0.03)	0.008
CRP (% difference)	2.3	(-6.9, 12.5)	6.2	(-3.9, 17.3)	0.25
IL-6 (% difference)	0.5	(-6.7, 8.3)	3.5	(-3.9, 11.5)	0.30
Year 15.4 risk factors					
SBP (mmHg)	-0.44	(-1.31, 0.43)	-0.63	(-1.63, 0.37)	0.22
DBP (mmHg)	-0.30	(-1.17, 0.56)	-0.14	(-1.05, 0.77)	0.75
Triglycerides (% difference*)	1.3	(-1.8, 4.6)	2.7	(-1.0, 6.5)	0.16
LDL-C (mmol/L)	0.01	(-0.04, 0.07)	0.04	(-0.01, 0.09)	0.13
HDL-C (mmol/L)	-0.02	(-0.04, 0.01)	-0.03	(-0.06, -0.00)	0.04
Glucose (mmol/L)	0.00	(-0.04, 0.04)	-0.01	(-0.06, 0.03)	0.50
Insulin (% difference*)	0.8	(-3.5, 5.4)	0.2	(-4.9, 5.6)	0.93
CRP (% difference*)	9.9	(-0.7, 21.7)	13.8	(3.2, 25.6)	0.01

25(OH)D, Total 25-hydroxyvitamin D; Apo-A1, Apolipoprotein-A1; Apo-B, Apolipoprotein-B; CRP, C-reactive protein; DBP, diastolic blood pressure; HDL-C, high-density lipoprotein cholesterol; IL-6, Interleukin-6; LDL-C, low-density lipoprotein cholesterol; SBP, systolic blood pressure

Associations are adjusted for maternal age at delivery, education level, pre-pregnancy BMI and smoking and physical activity during pregnancy, parity, socioeconomic position, ethnicity, and offspring gestational age at maternal 25(OH)D sampling, gender, and age and BMI at year 9.9 or 15.4 assessment

* Results are relative percentage differences in outcomes compared to the reference group (those with 25(OH)D>75 nmol/L)

† *P* for trend of difference in risk factors across 25(OH)D concentration groups

Supplemental table 5: Mean differences in offspring cardiovascular risk factors at mean ages 9.9 and 15.4 years in those with maternal 25(OH)D in pregnancy = 50 to 75 nmol/L (N=1,284) and those with maternal 25(OH)D < 50nmol/L (N=1,341), compared to those with maternal 25(OH)D > 75 nmol/L (N= 1,484), adjusted for potential confounders and offspring 25(OH)D in childhood

	Mean difference in outcomes comparing those with 25(OH)D=50 to75 nmol/L to those with 25(OH)D >75 nmol/L	95% CI	Mean difference in outcomes comparing those with 25(OH)D<50 to those with 25(OH)D >75 nmol/L	95% CI	*P*†
Year 9.9 risk factors					
SBP (mmHg)	0.34	(-0.40, 1.08)	0.59	(-0.14, 1.31)	0.08
DBP (mmHg)	0.14	(-0.39, 0.66)	0.44	(-0.05, 0.94)	0.05
Triglycerides (% difference)	-0.4	(-4.3, 3.7)	0.5	(-3.4, 4.5)	0.53
LDL-C (mmol/L)	0.01	(-0.04, 0.06)	0.05	(-0.01, 0.10)	0.11
HDL-C (mmol/L)	-0.004	(-0.03, 0.02)	-0.02	(-0.05, 0.01)	0.07
Apo-A1 (mg/dL)	0.00	(-0.02, 0.02)	-0.01	(-0.03, 0.01)	0.33
Apo-B (mg/dL)	0.01	(-0.01, 0.02)	0.02	(0.00, 0.03)	0.01
CRP (% difference)	2.3	(-6.9, 12.5)	6.2	(-3.9, 17.3)	0.12
IL-6 (% difference)	0.5	(-6.7, 8.3)	3.5	(-3.9, 11.5)	0.46
Year 15.4 risk factors					
SBP (mmHg)	-0.44	(-1.31, 0.43)	-0.63	(-1.63, 0.37)	0.16
DBP (mmHg)	-0.30	(-1.17, 0.56)	-0.14	(-1.05, 0.77)	0.84
Triglycerides (% difference*)	1.3	(-1.8, 4.6)	2.7	(-1.0, 6.5)	0.28
LDL-C (mmol/L)	0.01	(-0.04, 0.07)	0.04	(-0.01, 0.09)	0.27
HDL-C (mmol/L)	-0.02	(-0.04, 0.01)	-0.03	(-0.06, -0.00)	0.10
Glucose (mmol/L)	0.00	(-0.04, 0.04)	-0.01	(-0.06, 0.03)	0.41
Insulin (% difference*)	0.8	(-3.5, 5.4)	0.2	(-4.9, 5.6)	0.67
CRP (% difference*)	9.9	(-0.7, 21.7)	13.8	(3.2, 25.6)	0.02

25(OH)D, Total 25-hydroxyvitamin D; Apo-A1, Apolipoprotein-A1; Apo-B, Apolipoprotein-B; BMI, Body mass index; CRP, C-reactive protein; DBP, diastolic blood pressure; HDL-C, high density lipoprotein cholesterol; IL-6, Interleukin-6; LDL-C, low density lipoprotein cholesterol; SBP, systolic blood pressure

Associations are adjusted for maternal age at delivery, education level, pre-pregnancy BMI and smoking and physical activity during pregnancy, parity, socioeconomic position, ethnicity, and offspring gestational age at maternal 25(OH)D sampling, gender, age and BMI at year 9.9 or 15.4 assessment, and 25(OH)D concentration in childhood

* Results are percentage difference in risk factors compared to the reference group (those with 25(OH)D>75 nmol/L)

† *P* for trend of difference in risk factors across 25(OH)D concentration groups

Supplemental table 6: Associations of maternal 25(OH)D (unadjusted for season) measured in pregnancy with offspring cardiovascular risk factors measured at mean age 9.9 years and at mean age 15.4 years (N=4,109)

	Model 1			Model 2		
	Mean difference per 50nmol/L increase of 25(OH)D	95% CI	*P*	Mean difference per 50nmol/L increase of 25(OH)D	95% CI	*P*
9.9 year risk factors						
SBP (mmHg)	-0.35	(-0.76, 0.07)	0.10	-0.33	(-0.74, 0.08)	0.12
DBP (mmHg)	-0.23	(-0.54, 0.08)	0.14	-0.21	(-0.52, 0.10)	0.18
Triglycerides (% difference*)	0.1	(-2.3, 2.6)	0.91	-0.1	(-2.5, 2.4)	0.95
LDL-C (mmol/L)	-0.02	(-0.05, 0.01)	0.26	-0.02	(-0.06, 0.01)	0.21
HDL-C (mmol/L)	0.02	(-0.001, 0.03)	0.07	0.02	(-0.003, 0.03)	0.09
Apo-A1 (mg/dL)	0.01	(-0.004, 0.02)	0.18	0.01	(-0.01, 0.02)	0.34
Apo-B (mg/dL)	-0.01	(-0.02, -0.001)	0.03	-0.01	(-0.02, -0.001)	0.03
CRP (% difference*)	-5.1	(-10.3, 0.3)	0.06	-4.6	(-9.8, 1.0)	0.11
IL-6 (% difference*)	-3.0	(-7.4, 1.6)	0.20	-2.3	(-6.8, 2.4)	0.33
15.4 year risk factors						
SBP (mmHg)	0.46	(-0.17, 1.09)	0.15	0.45	(-0.18, 1.08)	0.16
DBP (mmHg)	0.14	(-0.45, 0.73)	0.64	0.13	(-0.46, 0.72)	0.67
Triglycerides (% difference*)	-1.2	(-3.5, 1.0)	0.29	-1.1	(-3.3, 1.2)	0.36
LDL-C (mmol/L)	-0.01	(-0.05, 0.02)	0.42	-0.02	(-0.05, 0.02)	0.35
HDL-C (mmol/L)	0.02	(-0.001, 0.03)	0.06	0.01	(-0.002, 0.03)	0.09
Glucose (mmol/L)	0.00	(-0.02, 0.03)	0.84	0.01	(-0.02, 0.03)	0.65
Insulin (% difference*)	0.1	(-3.2, 3.5)	0.96	0.7	(-2.6, 4.1)	0.70
CRP (% difference*)	-5.9	(-11.3, -0.1)	0.05	-5.9	(-11.4, -0.2)	0.04

25(OH)D, Total 25-hydroxyvitamin D; Apo-A1, Apolipoprotein-A1; Apo-B, Apolipoprotein-B; CRP, C-reactive protein; DBP, diastolic blood pressure; HDL-C, high density lipoprotein cholesterol; IL-6, Interleukin-6; LDL-C, low density lipoprotein cholesterol; SBP, systolic blood pressure

Model 1: adjusted for maternal age at delivery, education level, pre-pregnancy BMI and smoking and physical activity during pregnancy, parity, socioeconomic position, ethnicity, and offspring gestational age at maternal 25(OH)D sampling, gender, and age and BMI at year 9.9 or 15.4 assessment

Model 2: as model 1 plus offspring 25(OH)D concentration in childhood

* These outcomes were log transformed and differences represent a relative percent difference in the outcome per 50nmol/L increase of 25(OH)D.

Supplemental table 7: Associations of season-adjusted maternal 25(OH)D measured in pregnancy with offspring cardiovascular risk factors measured at mean age 9.9 years (N=2,099) and mean age 15.4 years (N=1,302) in the complete-case subsamples

	Model 1			Model 2		
	Mean difference per 50nmol/L increase of 25(OH)D	95% CI	*P*	Mean difference per 50nmol/L increase of 25(OH)D	95% CI	*P*
Year 9.9 risk factors						
SBP (mmHg)	-0.46	(-1.04, 0.12)	0.12	-0.43	(-1.01, 0.16)	0.15
DBP (mmHg)	-0.15	(-0.58, 0.27)	0.48	-0.12	(-0.54, 0.30)	0.58
Triglycerides (% difference*)	1.4	(-1.6, 4.4)	0.38	1.2	(-1.8, 4.3)	0.44
LDL-C (mmol/L)	-0.02	(-0.07, 0.02)	0.25	-0.03	(-0.07, 0.02)	0.23
HDL-C (mmol/L)	0.01	(-0.01, 0.03)	0.24	0.01	(-0.01, 0.03)	0.30
Apo-A1 (mg/dL)	0.00	(-0.01, 0.02)	0.72	0.00	(-0.01, 0.01)	0.94
Apo-B (mg/dL)	-0.01	(-0.02, 0.00)	0.07	-0.01	(-0.02, 0.00)	0.07
CRP (% difference*)	-6.5	(-13.1, 0.5)	0.07	-6.2	(-12.7, 0.9)	0.09
IL-6 (% difference*)	-4.7	(-10.1, 1.1)	0.11	-4.2	(-9.7, 1.6)	0.15
Year 15.4 risk factors						
SBP (mmHg)	-0.19	(-1.06, 0.67)	0.66	-0.26	(-1.13, 0.62)	0.56
DBP (mmHg)	0.34	(-0.39, 1.06)	0.37	0.29	(-0.45, 1.02)	0.44
Triglycerides (% difference*)	0.0	(-2.9, 3.0)	1.00	0.0	(-3.0, 3.0)	0.98
LDL-C (mmol/L)	-0.05	(-0.09, -0.00)	0.04	-0.05	(-0.09, -0.00)	0.03
HDL-C (mmol/L)	0.02	(-0.00, 0.04)	0.11	0.02	(-0.01, 0.04)	0.13
Glucose (mmol/L)	0.00	(-0.03, 0.03)	0.90	0.00	(-0.03, 0.03)	0.96
Insulin (% difference*)	0.3	(-3.4, 4.1)	0.89	0.9	(-2.9, 4.7)	0.66
CRP (% difference*)	-7.4	(-15.2, 1.1)	0.09	-7.0	(-14.9, 1.6)	0.11

25(OH)D, Total 25-hydroxyvitamin D; Apo-A1, Apolipoprotein-A1; Apo-B, Apolipoprotein-B; CRP, C-reactive protein; DBP, diastolic blood pressure; HDL-C, high density lipoprotein cholesterol; IL-6, Interleukin-6; LDL-C, low density lipoprotein cholesterol; SBP, systolic blood pressure

Model 1: adjusted for maternal age at delivery, education level, pre-pregnancy BMI and smoking and physical activity during pregnancy, parity, socioeconomic position, ethnicity, and offspring gestational age at maternal 25(OH)D sampling, gender, and age and BMI at year 9.9 or 15.4 assessment

Model 2: as model 1 plus offspring 25(OH)D concentration in childhood

* These outcomes were log transformed and differences represent a relative percent difference in the outcome per 50nmol/L increase of 25(OH)D.

References

1. Krishnaveni GV, Veena SR, Winder NR, et al. Maternal vitamin D status during pregnancy and body composition and cardiovascular risk markers in Indian children: the Mysore Parthenon Study. *Am J Clin Nutr,* 2011;**93**(3):628-35.
2. Gale CR, Robinson SM, Harvey NC, et al. Maternal vitamin D status during pregnancy and child outcomes. *Eur J Clin Nutr,* 2008;**62**(1):68-77.
3. Friedewald WT, Levy RI, Fredrickson DS. Estimation of the Concentration of Low-Density Lipoprotein Cholesterol in Plasma, Without Use of the Preparative Ultracentrifuge. *Clin Chem* 1972;**18**(6):499-502.
4. Liu J, Blair S, Teng Y, et al. Physical activity during pregnancy in a prospective cohort of British women: results from the Avon longitudinal study of parents and children. *European Journal of Epidemiology* 2011;**26**(3):237-47.
